# Soy and Breast Cancer: Focus on Angiogenesis

**DOI:** 10.3390/ijms160511728

**Published:** 2015-05-22

**Authors:** Lenka Varinska, Peter Gal, Gabriela Mojzisova, Ladislav Mirossay, Jan Mojzis

**Affiliations:** 1 Department of Pharmacology, P.J. Šafárik University, Faculty of Medicine, Trieda SNP 1, 040 11 Košice, Slovakia; E-Mails: lenka.varinska@upjs.sk (L.V.); peter.gal@upjs.sk (P.G.); ladislav.mirossay@upjs.sk (L.M.); 2 Department for Biomedical Research, East-Slovak Institute of Cardiovascular Diseases, Ondavská 8, 040 11 Košice, Slovakia; 3 Department of Pharmacognosy and Botany, Faculty of Pharmacy, Commenius University, Odbojárov 10, 832 10 Bratislava, Slovakia; 4 Institute of Anatomy, 1st Faculty of Medicine, Charles University, U nemocnice 3, 128 00 Prague, Czech Republic; 5 Department of Experimental Medicine, P.J. Šafárik University, Faculty of Medicine, Trieda SNP-1, 040 11 Košice, Slovakia; E-Mail: gabriela.mojzisova@upjs.sk

**Keywords:** soy, genistein, breast cancer, angiogenesis, galectins

## Abstract

Epidemiological studies have revealed that high consumption of soy products is associated with low incidences of hormone-dependent cancers, including breast and prostate cancer. Soybeans contain large amounts of isoflavones, such as the genistein and daidzain. Previously, it has been demonstrated that genistein, one of the predominant soy isoflavones, can inhibit several steps involved in carcinogenesis. It is suggested that genistein possesses pleiotropic molecular mechanisms of action including inhibition of tyrosine kinases, DNA topoisomerase II, 5α-reductase, galectin-induced G_2_/M arrest, protein histidine kinase, and cyclin-dependent kinases, modulation of different signaling pathways associated with the growth of cancer cells (e.g., NF-κB, Akt, MAPK), *etc*. Moreover, genistein is also a potent inhibitor of angiogenesis. Uncontrolled angiogenesis is considered as a key step in cancer growth, invasion, and metastasis. Genistein was found to inhibit angiogenesis through regulation of multiple pathways, such as regulation of VEGF, MMPs, EGFR expressions and NF-κB, PI3-K/Akt, ERK1/2 signaling pathways, thereby causing strong antiangiogenic effects. This review focuses on the antiangiogenic properties of soy isoflavonoids and examines their possible underlying mechanisms.

## 1. Introduction

Breast cancer is the second most frequently diagnosed cancer and the leading cause of death among woman in developed countries, accounting for 22% of new cases each year [1]. Data from human studies have shown great differences in breast cancer incidence among woman with Western lifestyles (e.g., United States and Western Europe) and woman from Asia (e.g., Japan and Chinese) [2]. Results from epidemiological studies suggest that diet may strongly contribute to this variation in the breast cancer prevalence [3,4,5]. One possible explanation of this phenomenon is a high consumption of soy foods in Asia [6]. It is suggested that in Asian countries the average daily intake of soy isoflavones is 25–50 mg [7], while in the U.S. and Europe is less than 1 mg [8]. Ability of soy to reduced risk of breast cancer in Chinese women was for the first time documented by Lee and coworkers [9]. So far, several epidemiologic studies evaluating the relation between soy intake and breast cancer incidence has been conducted in the world [10,11,12,13,14]. Although all of these studies had some limitations, it is generally accepted that high soy intake is associated with reduced risk of breast cancer in Asian women.

Soybeans contain several constituents able to modulate carcinogenesis, namely initiation, promotion and cancer progression [15,16]. Among them, genistein and other phytoestrogens attract great interest, as they exhibit a plethora of biological actions, including breast and prostate cancer chemopreventive activity [17,18,19,20,21].

It is well known that many risk factors for breast cancer are related to prolonged exposure to estrogen and other hormones. Furthermore, most breast cancers are hormone-receptor-positive [22]. Soy phytoestrogens, such as genistein, daidzein, and glycitein, are isoflavonoids closely related to human 17β-estradiol [23], but with lower estrogenic activity [24]. Genistein has been identified as the predominant isoflavone in soybean. It possesses biphasic effect on estrogen receptor (ER) positive breast cancer cells. At low concentrations, it stimulates growth of ER positive breast cancer cells, whereas at higher concentrations growth of breast cancer cells is inhibited [25,26]. Apart from its estrogenic/antiestrogenic activity, genistein inhibits also growth of hormone non-dependent cancer cells [27,28]. Based on available experimental data, it is suggested that genistein can influence mechanisms involved in cell proliferation and possesses pleiotropic molecular mechanisms of action. Genistein is a well-known RTKs inhibitor, which may prevent abundant cell proliferation or abnormal angiogenesis by inhibiting receptor-associated tyrosine kinases (RTK)-mediated signaling pathways [29]. Furthermore, genistein inhibits DNA topoisomerase II activity [30] and is able to suppress its expression [31]. Soy isoflavonoids exert different anti-cancer mechanisms: (i) inhibition of 5α-reductase, protein histidine kinase, and cyclin-dependent kinases [32,33,34,35]; (ii) decrease the signaling pathways associated with the growth of cancer cells (e.g., NF-κB, Akt, MAPK); and (iii) apoptosis induction [36].

Taken together, genistein and other soy isoflavonoids exhibit multiple effects on the human malignant cells and also modulate selected steps of angiogenesis.

## 2. Angiogenesis and Breast Cancer

During embryonic development, endothelial progenitor cells (angioblasts) form a primitive vascular network of small capillaries in a process termed “vasculogenesis”. Subsequent vessel sprouting (angiogenesis) from pre-existing vessels results in the creation of arteries and veins [37]. Angiogenesis is not temporally restricted to embryonic development, but is also active under specific physiological conditions in healthy adults. This process is regulated by a wide range of angiogenic inducers (e.g., growth factors, chemokines, angiogenic enzymes, endothelial-specific receptors, and adhesion molecules) as well as various endogenous angiogenesis inhibitors, like angiostatin, endostatin, thrombospondin, and canstatin. Imbalances between the angiogenic inducers and inhibitors may result in pathologies, such as arthritis, psoriasis, obesity, asthma, atherosclerosis, heart and brain ischemia, neurodegeneration, hypertension, pre-eclampsia, respiratory distress, osteoporosis, and many other disorders [38]. Angiogenesis has also been recognized as a basic prerequisite for progression, proliferation and metastatic spread of tumors.

During tumorigenesis, most tumors start growing as avascular nodules to a certain size not exceeding a few millimeters. This first phase in tumor expansion is followed by the switch from avascular to vascular phenotypes due to the malignant tissue hypoxia and malnutrition. The onset of the “angiogenic switch” is a discrete step in the development of malignant tumors that is inevitable for growth and metastasis [39]. Although it has been recognized for many centuries that neoplastic tissue is more vascular than its normal counterpart, it is only since Folkmans’ hypothesis on anti-angiogenesis that extensive research in the regulation of angiogenesis has started [40]. The vascular endothelial growth factor (VEGF) family represents the most important component in the angiogenic pathway [41]. The most intensively studied member of VEGF family is VEGF-A. It is considered the most important regulator in human physiologic and pathologic angiogenesis and high levels of circulating VEGF are a well-established indicator of poor prognosis in several cancers, including breast cancer [42,43]. A monoclonal antibody designed against VEGF-A (bevacizumab) was the first U.S. Food and Drug Administration (FDA)-approved anti-angiogenic drug.

Despite showing efficacy in some cancer types (such as metastatic colorectal cancer or non-squamous non-small cell lung cancer), conflicting results about the benefit of VEGF blockade have been obtained in advanced breast cancer [44]. The E2100 phase III study demonstrated that the combination of bevacizumab with paclitaxel was shown to result in superior progression-free survival (PFS) and increased the objective response rate compared to chemotherapy alone [45]. Based on these, data bevacizumab was approved by the European Medicines Agency (EMEA) and the FDA for the treatment of HER2-negative metastatic breast cancer when administered in combination with chemotherapy. In another three studies (AVADO, RIBBON-1, and RIBBON-2), despite the improvement in PFS and the proportion of patients achieving a response, the combination of bevacizumab and chemotherapy has so far proven disappointment in term of improving overall survival (OS) [46,47,48]. As a consequence of this, and increase in incidence of adverse events in bevacizumab/chemotherapy arm, the FDA withdrew its earlier approval in this indication. A new suggested approach how to maximize the benefit of bevacizumab and restrict tumor re-growth after anti-angiogenic drugs is sustained VEGF suppression, as it was validated in metastatic colorectal cancer [49,50]. Adherence to a maintenance therapy after first-line treatment appears to be effective also in breast cancer. TANIA study was designed to evaluate further bevacizumab in bevacizumab-pretreated locally recurrent/metastatic breast cancer [51]. The analyses of data showed a benefit from the use of bevacizumab across multiple lines of therapy. Continued bevacizumab with capecitabine statistically significant and clinically meaningful improved PFS and OS in patients benefiting from first-line bevacizumab-containing therapy what was observed in the open-label randomized phase III IMELDA trial [52]. Thus, prolonging first-line chemotherapy with maintenance treatment may influence OS.

Another angiostatic drugs, tyrosine kinase inhibitors, namely sorafenib, sunitinib, pazopanib and axitinib, have remained ineffective in treating breast cancer [53,54,55] despite the benefit showed in other cancers [56]. Everolimus, a small molecule inhibitor of mammalian target of rapamycin (mTOR) and recombinant human endostatin are other anti-angiogenic molecules that undergo clinical trials and might be used in treatment settings of breast cancer [57,58]. Selected clinical trials are presented in Table 1.

In conclusion, according to the present results, the role of anti-angiogenic drugs still remain unclear in breast cancer and needs further investigation. Alternative strategies, based on targeting of microRNAs, may serve as novel therapeutic targets [59]. Likewise, phytotherapy could offer many new opportunities in treating breast cancer patients. It is commonly used in many fields of medicine due to its excellent properties, such as simple preparation and administration as well as poor presence of side effects and acceptable efficiency. The use of natural products may represent a feasible option of cancer-treatment in many regions of the world. It has been shown that almost 80% of the world’s population uses medicine of herbal origin for primary health care. Accordingly, the World Health Organization has also recommended natural agents as an alternative to synthetic pharmaceuticals in developed countries [60].

**Table 1:** Antiangiogenic drugs used in breast cancer treatment-clinical trials.

Breast Cancer	Treatment	Clinical Trial	Outcame	reference
**Neoadjuvant**	doxorubicin/docetaxel/cyclophosphamide ± bevacizumab	NCT00408408	improvement in pCR DFS and OS-data under way	[61]
	epirubicin-cyclophosphamide/docetaxel ± bevacizumab	NCT00567554	no improvement in DFS and OS improvement in pCR	[62,63]
**Adjuvant**	anthracycline, taxane or both ± bevacizumab	BEATRICE	no improvement in DFS, OS	[64]
	adjuvant hormone therapy ± everolimus	NCT01805271	ongoing trial	-
**First-line**	paclitaxel ± bevacizumab	E2100	improvement in PFS not OS	[45]
	docetaxel ± bevacizumab	AVADO	improvement in PFS not OS	[46]
	capecitabine/taxane/anthracycline based chemotherapy ± bevacizumab	RIBBON-1	improvement in DFS not OS	[53]
	trastuzumab, docetaxel ± bevacizumab	AVEREL	no improvement in PFS or OS	[65]
	Docetaxel ± sunitinib	NCT00393939	no improvement in PFS or OS	[54]
**First-line/second-line**	capecitabine ± sorafenib	NCT01234337	ongoing study	[66]
**Second-line**	capecitabine ± bevacizumab	AVF2119	no improvement in PFS or OS	[67]
	capecitabine ± sunitinib	NCT00435409	no improvement in PFS or OS	[55]
	exemestane ± everolimus	NCT00863655	improvement in PFS not OS	[57]
	trastuzumab, vinorelbine ± everolimus	NCT01007942	improvement in PFS, OS in progress	[68]
	capecitabine/taxane/gemcitabine/vinorelbine based chemotherapy ± bevacizumab	RIBBON-2	improvement in PFS not OS	[48]
	capecitabine *vs.* sunitinib	NCT00373113	inferior PFS and OS for sunitinib arm	[69]
	chemotherapy ± bevacizumab	TANIA	improvement in PFS, OS not reported	[51]
	bevacizumab + capecitabine bavacizumab alone	IMELDA	improvement in PFS and OS	[52]

pCR pathological complete response; OS overall survival; DFS disease free survival; PFS progression free survival.

## 3. Antiangiogenic Effect of Soy Isoflavonoids

Formation of new blood vessels occurs as a result of several processes, including activation of endothelial cells, destruction of matrix by proteolytic enzymes, migration and proliferation of endothelial cells as well as formation of tubular structures [70]. Tumor angiogenesis can be inhibited by blocking some of these steps. Several papers have been published referring on the modulatory effect of flavonoids on angiogenesis [71,72,73,74,75,76,77,78]. Soy isoflavonoids also exhibit anti-angiogenic activities, but the precise mechanism of inhibition remains unclear. These compounds exert anti-angiogenic effect either directly through endothelial cells (EC) or indirectly by modulating the tumor microenvironment [79,80,81].

As mentioned above, VEGF is an important regulator of angiogenesis and inhibition of VEGF secretion or blockade of its receptors is associated with suppression of blood vessels formation [82]. Genistein at doses 5–50 µM prevented the growth of human umbilical vein endothelial cells (HUVECs) after stimulation with VEGF. Moreover, genistein (10–50 µM) significantly inhibited basal VEGF expression and hypoxia-stimulated VEGF expression in both cancer cells and HUVECs. Expression of the VEGF receptor fms-like tyrosine kinase-1 in HUVECs was also reduced after treatment with genistein. As authors suggested, genistein may inhibit tumor angiogenesis through the suppression of VEGF-mediated signaling pathways between tumor cells and vascular endothelial cells [83]. Loss of VEGF activity under hypoxic condition after treatment with genistein may also be associated with ability of genistein to interfere with the post-transcriptional induction of VEGF by hypoxia [84]. Later, Yu and co-workers [85] studied the effect of genistein on VEGF secretion and Vegf mRNA expression in mammary cancer cells. They found that the level of VEGF protein in genistein-treated cells was significantly decreased compared with non-treated cells. Furthermore, the level of VEGF mRNA expression was consistent with the alteration of level of protein expression.

Anti-angiogenic effect of genistein has also been reported by Su and co-workers [86]. They showed a dose-dependent inhibition of expression/excretion of VEGF. Genistein also decreased VEGF mRNA expression both under normoxic and hypoxic conditions. Similarly, lower levels of VEGF mRNA were found in xenograft tumors. Moreover, activation of hypoxia inducible factor-1 (HIF-1) was impaired in cells treated with genistein under hypoxic conditions. As it is suggested, anti-angiogenic effect of genistein can be mediated by the inhibition of the HIF-1 activation with subsequent inhibition of VEGF gene expression [87]. A similar relation between VEGF and HIF-1 was also recently documented by Aditya *et al.* [88]. They found that treatment of cancer cells with combination of curcumin and genistein led to angiogenesis inhibition by acting on VEGF protein expression via down regulation of HIF-1α and aryl hydrocarbon receptor nuclear translocator. Genistein, in addition to VEGF mRNA suppression, at a low physiological dose (2.5 µmol/L) also affected the levels of mRNA for VEGF receptor 1 (VEGFR-1) and 2 (VEGFR-2) in HUVECs [89]. Furthermore, in various *in vivo* experiments (using xenografts, chick chorioallantoic membrane or zebrafish experimental models), genistein significantly reduced microvessel density [90,91,92]. Moreover, other pro-angiogenic factors such as platelet-derived growth factor (PDGF), tissue factor (TF), urokinase plasminogen activator (uPA), matrix metalloproteinase-2 and -9 (MMP-2, and MMP-9) were also inhibited in genistein-treated cells [86]. On the other hand, up-regulation of anti-angiogenic factors (e.g., plasminogen activator inhibitor-1, endostatin, angiostatin, and thrombospondin-1) was observed [86]. On the contrary, no significant angiogenesis inhibition was noticed for daidzein.

Components of the extracellular matrix (ECM) play pivotal roles in docking cells and engaging them in the complex molecular interplay. Aberrations in this complex network, either engineered in mice or detected in patients, can lead to diseases [93,94]. Constituents of the ECM have proven capable to stimulate angiogenesis [95]. Lysis of ECM is necessary to promote endothelial cell invasion and sprouting. The most relevant proteolytic enzymes involved in angiogenesis are MMP-2 and -9 and uPA-plasmin system [96,97,98]. Genistein can interfere with the activity of MMPs, reducing the degradation of ECM, which forms the basis of angiogenic switch. Treatment of HUVECs with VEGF/bFGF (basic fibroblast growth factor) caused significant increase in MMP-1 production as well as induction of pro-MMP-2 activation [99]. However, pretreatment with genistein completely prevented the VEGF/bFGF-stimulated increase in both MMP-1 expression and pro-MMP-2 activation. Moreover, genistein also blocked VEGF/bFGF-induced uPA and PA inhibitor-1 expression. Later, Kumi-Diaka *et al.* [100] also documented inhibition of MMP-2 expression in cells treated with genistein. Additionally, genistein reduced the mRNA level of several MMPs including MMP-2, MMP-3, MMP-13, and MMP-15 [101]. However, not all studies confirmed ability of genistein to block activity of MMPs. Farina *et al.* [102] found no effect of genistein on MMP-2 and MMP-9 activity. On the other hand, *in vivo* administration of either genistein or a soy-based diet reduced tumor-induced angiogenesis in syngeneic mice implanted with B16 or F3II cells.

Recent application of genome-wide screening revealed that genistein or daidzein down-regulated a set of genes necessary for the angiogenesis pathway either in HUVECs or cancer cells. In Huvecs, Piao *et al.* [103] studied effect of genistein (10.0 µmol/L) on expression of several genes involved in cell proliferation, adhesion, transcription, translation, metabolism, cytoskeleton or apoptosis. Genistein was observed to down-regulate cell adhesion-related genes (e.g., VE-cadherin, integrin αV, connexin 43, and multimerin) on mRNA level. In the study of Rabiau *et al.* [104] the main result showed a down-regulation of epidermal growth factor (EGF) and insulin-like growth factor 1 (IGF-1) after treatment with genistein (40.0 µmol/L) or daidzein (110.0 µmol/L). Moreover, expression of other pro-angiogenic molecules such as cadherin 5, PDGF, VEGF, fibroblast growth factor 1 (FGF 1), MMP-9, uPA, angiopoietin 2, hepatocyte growth factor (HGF), and interleukin 18 were also significantly down-regulated in cancer cells treated with genistein or daidzein, respectively. Furthermore, genistein has also been observed to down-regulate gene expression of VEGF, uPA receptor, lysophosphatidic acid receptor [105] as well as fibronectin and MMP-13 [106].

Cancer cells are known to have alterations in multiple cellular signaling pathways. A number of studies suggest that chemopreventive effects of genistein are due to the regulation of different important cellular signaling pathways [107]. It has been accepted that nuclear factor κB (NF-κB) signaling pathway plays an important roles in physiological as well as pathological processes such as control of cell growth, apoptosis, inflammation, invasion, transformation and angiogenesis [108]. Thus, inhibition of NF-κB activity in cancer/endothelial cells may provide a target for cancer treatment and/or prevention. Recently, Wang *et al.* [109] have found that genistein suppressed MMP-9 transcription by inhibiting NF-κB activity. It suppressed 12-*O*-tetradecanoylphorbol-13-acetate (TPA)-induced NF-κB—specific DNA-protein binding compared to TPA via inhibition of NF-κB nuclear translocation through inhibitor of kappa B inhibitory signaling pathways. Moreover, genistein also suppressed TPA-induced activation of extracellular signal-regulated kinases/phosphatidylinositol 3-kinase/protein kinase B (ERK/PI3K/Akt) upstream of NF-κB.

Furthermore, consumption of genistein significantly down-regulated cell proliferation as well as receptors for growth factors (EGFR, IGF-1R) and mitogen-activated protein kinase (MAPKs) that play a significant role in stimulating cell proliferation (ERK-1/2) in prostates of TRAMP mice [110]. Recently, Yu *et al.* [111] studied effect of genistein on VEGF-stimulated HUVECs. Genistein at concentrations 1.0–100.0 µmol/L effectively inhibited VEGF-induced protein tyrosine kinase stimulation. Simultaneously, levels and activity of MMP-2 and -9 were significantly suppressed in genistein-treated HUVECs. Exposure of HUVECs to genistein also reduced VEGF-mediated phosphorylation of c-Jun N-terminal kinases (JNK) and p38. In contrast to the previous study [90], genistein did not significantly decrease endothelial cell ERK-1/2 phosphorylation. In another study, Huang *et al.* [112] also showed ability of genistein to inhibit MMP-2 activity as well as its ability to block activation of p38 MAPK by transforming growth factor β (TGF-β). In addition to this study, genistein has been found to block TGF-β-induced activation of MMP-2 and p38 MAPK via blocking activation of the MAP kinase activated protein kinase 2 and the 27-kDa heat shock protein a down-downstream effectors of p38 MAP kinase [113].

Prostaglandins play a key role in many of physiological as well as pathological processes. They are generated from arachidonate by the action of cyclooxygenase (COX). Cyclooxygenase-2 (COX-2), a COX isoenzyme catalyzing the production of prostaglandins, is reported to be involved in the pathogenesis of many human tumors [114,115]. Some results also indicate that COX-2 is associated with increased VEGF production and angiogenesis [116,117,118]. Furthermore, COX-2 has been shown to enhance bFGF-induced angiogenesis through induction of VEGF in rat sponge implants [119]. The results of Akarasereenont *et al.* [120] showed that COX-2 is over-expressed in HUVECs treated with VEGF. Increase in both protein level and COX-2 activity was significantly reduced when cells were co-incubated with genistein. This effect was strongly correlated with inhibition of VEGF-associated protein tyrosine kinase.

Another possible field of genistein action is cancer-related inflammation. The association between inflammation and cancer is now accepted as enabling characteristic of cancer [121]. Inflammation in the tumor microenvironment affects many aspects of malignancy. It aids in the growth and survival of malignant cells, stimulates angiogenesis and suppresses adaptive immune responses [122,123]. Prostaglandins produced by COX-2 play an important role in inflammation, thus inhibition of this COX isoform is suggested to be potential target for cancer chemoprevention/treatment [124,125,126]. In the study of Hwang *et al.* [127], the effect of genistein on TPA-induced inflammation-related signaling pathway was studied. Genistein alone or in combination with capsaicin efficiently reduced COX-2 expression in MCF-7 cells. This effect was associated with activation of AMP-activated protein kinase. Ability of genistein or daidzein to inhibit COX-2 expression as well as activation of NF-κB in TPA treated animals was documented also by others authors [128,129,130].

Another important inflammatory component of the stroma of many tumors are tumor-associated macrophages (TAMs). They play a key role in tumor angiogenesis by secreting a numerous of substances that promote angiogenesis including VEGF, PDGF, TGF-β, FGF, MMP-2, MMP-7, MMP-9, MMP-12, COX-2 as well as several chemokines [131,132,133,134]. It was demonstrated that macrophage-released VEGF in solid tumors contributed to the initiation of tumor angiogenesis with an increased number of vessels and branches [135]. Thus, targeting of TAMs may represent a new strategy to complement “classical” anticancer therapy. Genistein was found to reduce the number of TAMs by 96% when administered to rats bearing MAT-Lu tumors. Moreover, genistein in *in vitro* conditions significantly suppressed tumor necrosis factors-α secretion and lipopolysaccharide-stimulated production of granulocyte-macrophage colony-stimulating factor, both pro-angiogenic factors [136].

Glyceollins, a novel class of soybean phytoalexins with potential cancer-preventive effects, also possesses antiangiogenic activity. Lee *et al.* [137] found that glyceollins *in vitro* inhibited VEGFR-2 and FGFR-1 activity and their downstream signaling pathways (e.g., ERK1/2, JNK). Glyceollins also significantly suppressed microvessel density in both *in vivo* and *ex vivo* conditions. Later, these authors studied effect of glyceollins on HIF-1α regulation. Under hypoxic conditions they reduced the expression of HIF-1α in various cancer cells by inhibiting the PI3K/AKT/mTOR pathway. This effect may also be associated with blockade of the interaction of HIF-1α with 90-kDa heat shock protein. Moreover, glyceollins inhibited the expression of HIF-1α-induced genes such as the VEGF. Furthermore, glyceollins were found to decrease microvessel density in solid tumor tissues [138]. Additionally to previously mentioned articles, glyceollins were documented to inhibit the transcriptional activation of COX-2 by regulating NF-κB activity. Based on these results, it may be hypothesized that glyceollins may modulate the cancer inflammatory microenvironment [139].

### Do Galectins Mediate Estrogen-Dependent Signals Following Soy Consumption?

Increasing attention is given to the role of glycosylation of proteins and lipids where the sugar-encoded information is being translated into several cellular activities by endogenous lectins [140,141]. Since galectins play an important role in the processes of cell proliferation, differentiation, migration and extracellular matrix formation [142,143], it has been proven that they are significant modulators of the tumor/wound microenvironments [144]. In general, it has been shown that the main role of galectin-1 in cancer progression represents its immunosuppressive effect, which facilitates pro-tumorigenic microenvironment [145,146] and stimulates angiogenesis [147]. Similarly, over-expression of galectin-3 in hypoxic breast tumors was associated with the presence of more aggressive tumors leading to a poor prognosis [148,149]. Galectin-3 is also an important mediator of VEGF- and bFGF-mediated angiogenic response [150].

Several studies have focused on the soy compound isoflavonoid genistein and its potential chemopreventive activities in the breast cancer [151]. It was shown that galectin-1 is up-regulated in invasive breast carcinoma demonstrating a positive correlation with the TNM staging system [152]. Accordingly, silencing of galectin-1 in breast carcinoma model inhibited tumor growth and prevented metastatic disease [145]. Soy consumption reduced galectin-1 intensities in blood mononuclear cells [153]. Moreover, our results indicated that the estrogen receptor-α agonist increases the expression of galectin-1 in keratinocytes (unpublished data). Since long-term estradiol deprivation enhanced estrogen sensitivity in breast cancer, it may by suggested that soy consumption could overcomes tumor-associated-galectin-1-induced immunosuppression [154].

Similarly, at the cellular level, it was well demonstrated that the phytoestrogen genistein inhibits proliferation of human breast carcinoma cell lines [151]. It has been shown that the genistein-induced G_2_/M arrest was mediated via galectin-3 [155]. Contrary to previously published reports, this phytoestrogen acted as a key regulator in the WNT/β-catenin signaling pathway [156], which led to endothelial cells tube-like formation on Matrigel [157]. Moreover, genistein stimulated the growth of estrogen receptor-positive breast cancer cells MCF-7 in an athymic mice xenograft model [158].

**Figure 1:**
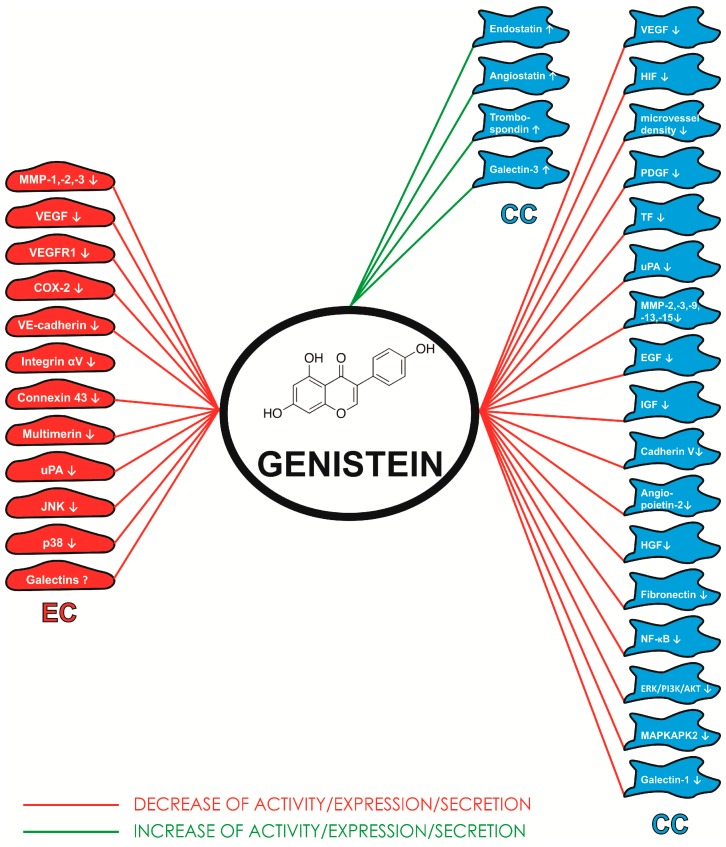
Molecular targets of genistein on endothelial (EC) and cancer (CC) cells. Akt-protein kinase B; bFGF—basic fibroblast growth factor; COX-2—cyclooxygenase-2; EGF—fibroblast growth factor; ERK—extracellular signal-regulated kinases; HIF—hypoxia inducible factor; IGF—insulin-like growth factor; JNK-c—Jun *N*-terminal kinases; MAPK—mitogen-activated protein kinase; MAPKAPK2—MAP kinase activated protein kinase 2; MMP-matrix metalloproteinase; NF-κB—nuclear factor κB; PDGF—platelet-derived growth factor; TF—tissue factor; TNF-α—tumor necrosis factors α; uPA—urokinase plasminogen activator; VEGF—vascular endothelial growth factor; VEGFR1—receptor for vascular endothelial growth factor 1.

From this point of view, the intriguing relationships between estrogens and galectins need to be studied in detail to avoid complications in patient treatment. Of note, data on the relationships between other galectins and estrogens in the breast cancer and/or angiogenesis has not yet been documented. Nevertheless, data summarized in this review could have broad implications for developing novel, estrogen/carbohydrate-based therapeutic agents for inhibition of tumor growth and angiogenesis. Figure 1 summarizes the molecular targets of genistein on endothelial and cancer cells.

## 4. Conclusions

In conclusion, anticarcinogenic effects of soy isoflavonoids, particularly genistein, are mediated via several molecular pathways. In addition to the direct effects of isoflavonoids on cancer cells, genistein also modulates selected steps of angiogenesis namely growth and sprouting of endothelial cells, microcapillary tube formation and/or inhibition of several cell signaling pathways. In *in vivo* experiments, genistein-induced growth suppression of experimental tumors was also associated with significant reduction of microvessel density.

Although more studies are required to fully elucidate the mechanism of antiangiogenic action of soy isoflavones, and more clinical trials are needed to validate their usefulness in clinical practice, experimental data have shown that genistein is a promising agent for cancer chemoprevention.

## Supplementary Files

Supplementary File 1
