# Supplementary material for: Soy and Breast Cancer: Focus on Angiogenesis
Source: Int J Mol Sci. 2015 May 22;16(5):11728–49. doi: 10.3390/ijms160511728 (PMC4463727; doi:10.3390/ijms160511728)
Supplement: Supplementary File 1 [file ijms-16-11728-s001.pdf]

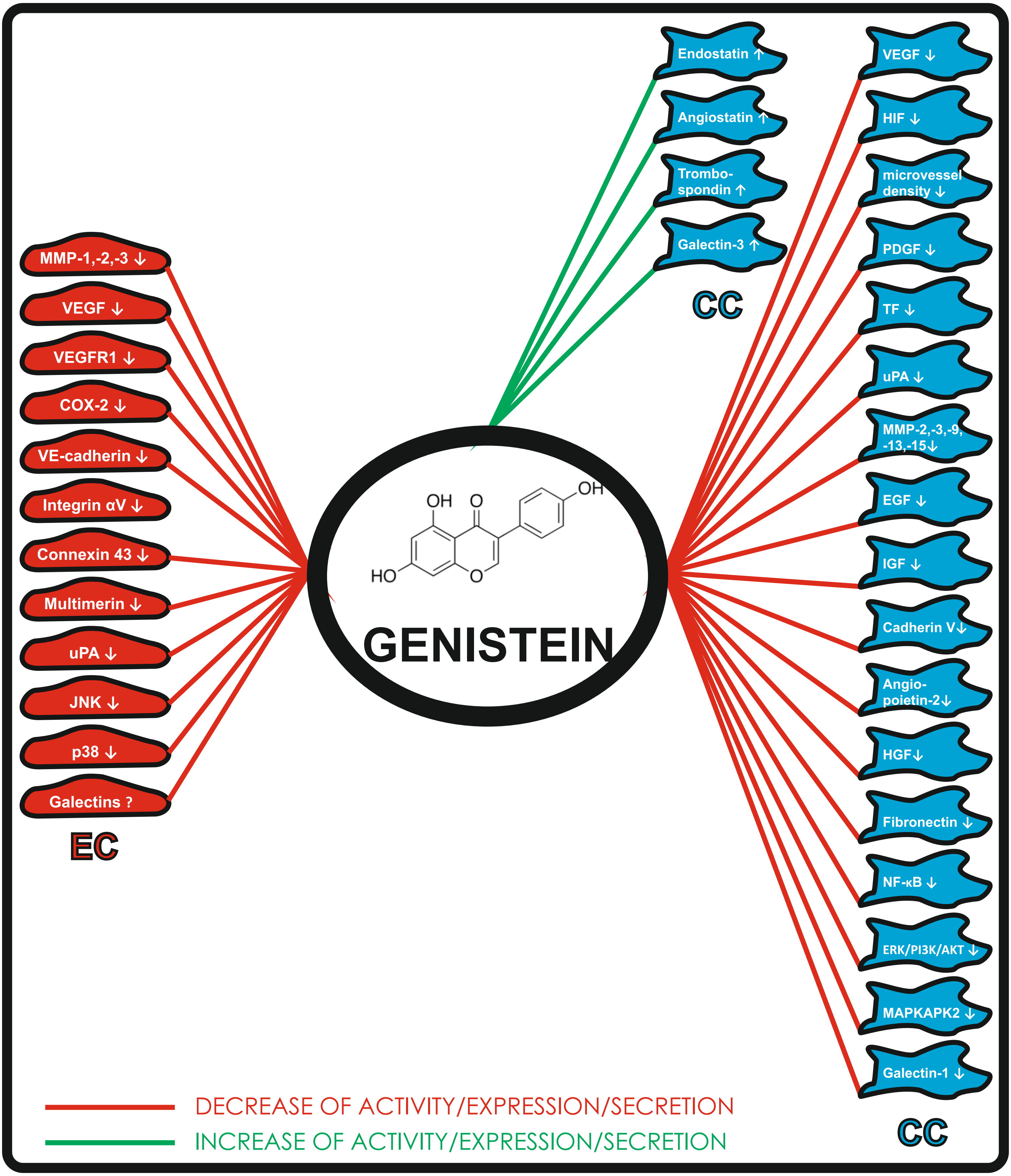

Endostatin ↑
Angiostatin ↑
Trombo-spondin ↑
Galectin-3 ↑
CC
VEGF ↓
HIF ↓
microvessel density ↓
PDGF ↓
TF ↓
uPA ↓
MMP-2,-3,-9,-13,-15↓
EGF ↓
IGF ↓
Cadherin V↓
Angio-poietin-2↓
HGF↓
Fibronectin ↓
NF-κB ↓
ERK/PI3K/AKT ↓
MAPKAPK2 ↓
Galectin-1 ↓
CC
MMP-1,-2,-3 ↓
VEGF ↓
VEGFR1 ↓
COX-2 ↓
VE-cadherin ↓
Integrin αV ↓
Connexin 43 ↓
Multimerin ↓
uPA ↓
JNK ↓
p38 ↓
Galectins ?
EC
OH
O
OH
HO
O
GENISTEIN
DECREASE OF ACTIVITY/EXPRESSION/SECRETION
INCREASE OF ACTIVITY/EXPRESSION/SECRETION
